# Immune network dysregulation in rheumatoid arthritis and systemic lupus erythematosus: cytokine signatures, autoantibody profiles, and implications for precision medicine

**DOI:** 10.3389/fmed.2026.1841343

**Published:** 2026-07-16

**Authors:** Benjuan Wu, Liya Zhang, Fuxi Chen

**Affiliations:** 1 Rheumatology and Immunology Department, Tianjin First Central Hospital, Naikai, Tianjin, China; 2 The Second Department of General Medicine, Tianjin First Central Hospital, Naikai, Tianjin, China; 3 Department of Surgery, Beichen Hospital of Nankai University, Tianjin, China

**Keywords:** autoantibodies, cytokine signatures, immune network dysregulation, immune sub-phenotype, precision medicine, rheumatoid arthritis, systemic lupus erythematosus

## Abstract

**Background:**

Rheumatoid arthritis (RA) and systemic lupus erythematosus (SLE) are systemic autoimmune diseases characterized by chronic inflammation, immune dysregulation, and heterogeneous clinical manifestations. Despite differences in target organs, both share overlapping pathogenic mechanisms, such as cytokine imbalance and autoantibody production. However, integrated evaluation of cytokine signatures and autoantibody profiles remains limited, limiting their translational application in precision medicine.

**Aim:**

This study aimed to characterize immune network dysregulation in RA and SLE using an integrated cross-disease analytical framework combining cytokine signatures, autoantibody profiles, network-based immune analysis, and immune sub-phenotype stratification, and to evaluate their translational relevance for precision medicine.

**Methodology:**

This retrospective observational study included 875 patients (RA: *n* = 450; SLE: *n* = 425) diagnosed between January 2021 and December 2025. Cytokine levels were measured using multiplex immunoassays and normalized to z-scores, while validated assays were used to determine autoantibody profiles. Correlation analysis, multivariate regression, principal component analysis (PCA), and hierarchical clustering were applied to identify immune interactions and sub-phenotypes. Statistical analyses were performed using SPSS and R, with adjustment for confounders and multiple testing.

**Results:**

Distinct, disease-specific immune signatures were observed. Pro-inflammatory cytokines, particularly IL-6 and IL-17, were significantly elevated in RA and were positively correlated with disease activity (*r* = 0.48 and *r* = 0.42, respectively). In SLE, interferon-related and regulatory cytokines, including IFN-*γ* and MCP-1, showed stronger associations with disease activity and organ involvement (*r* = 0.51 and *r* = 0.52). Autoantibody profiles differed: RF and anti-CCP were predominant in RA, whereas SLE exhibited greater autoantibody diversity and multi-positivity (61.5%). Network analysis revealed IL-6–CRP interactions in RA and IFN-*γ*–autoantibody burden interactions in SLE. PCA identified IL-6, IFN-γ, MCP-1, and autoantibody burden as key drivers of immune variability. Clustering defined five immune sub-phenotypes with distinct clinical outcomes, such as high-inflammatory RA and high-risk SLE flare clusters. Cytokines emerged as independent predictors of disease activity, whereas autoantibody burden showed limited predictive value (*p* = 0.08).

**Conclusion:**

Integrated immune profiling highlights both shared and distinct immune dysregulation in RA and SLE. Cytokine signatures outperform autoantibody burden in predicting disease activity and outcomes, supporting their role as key biomarkers for patient stratification and precision medicine.

## 1 Introduction

Rheumatoid arthritis (RA) and systemic lupus erythematosus (SLE) are prototypical systemic autoimmune diseases characterized by chronic inflammation, multi-organ involvement, and heterogeneous clinical manifestations. Although the major affected tissues in both RA and SLE are different, namely the joints in RA and multiple organ systems in SLE, both disorders have common pathophysiologic mechanisms of immune dysregulation, such as cytokine signaling dysregulation, production of autoantibodies, and the disruption of immune cell networks (1, 2). These common pathogenic pathways emphasize the possibility of shared molecular signatures that could guide precision medicine.

Cytokines are the key mediators of immune responses, coordinating innate and adaptive immune responses. Imbalanced cytokine networks are associated with sustained inflammatory processes in RA and SLE, and tissue destruction and disease exacerbation are linked to pro-inflammatory factors such as TNF-*α*, IL-6, and IFN-α (3, 4). As shown by Duarte-Delgado et al. (1), there are distinct patterns of cytokines between RA and SLE, with correlations between specific cytokine patterns and clinical variables, such as disease activity scores and hematologic variables. In line with the findings, Khatri et al. (4) highlighted the critical role of type I interferons in the pathogenesis of SLE, emphasizing their role in systemic autoimmunity by linking B-cell hyperactivation and autoantibody formation.

Another hallmark of aberrant immune activation is the presence of autoantibodies. Rheumatoid factor (RF) and anti-citrullinated protein antibodies (ACPA) are also diagnostic and pathogenic in RA, which is associated with joint erosion and inflammatory processes in the synovium (5). The presence of a broader range of autoantibodies in SLE, such as anti-dsDNA, anti-Sm, and anti-Ro/SSA, indicates systemic immune disruption and is associated with organ-specific manifestations (6). Additional autoantibodies implicated in SLE pathogenesis, including antiphospholipid antibodies, anti-ribosomal P, anti-chromatin, anti-SmRNP, lupus anticoagulant, and anti-β2-glycoprotein-I antibodies, further reflect the complexity and heterogeneity of humoral immune dysregulation in systemic autoimmunity. Recent research suggests that such autoantibody patterns are strongly correlated with specific cytokine signatures, indicating that the combined study of humoral and soluble immune mediators can provide a mechanistic understanding of disease heterogeneity (7, 8).

In addition to immune mediators, there are metabolic intersections with immune dysregulation in autoimmune diseases. SLE pathogenesis has also been associated with immune-metabolic remodeling, including altered glycolysis and mitochondrial activity, which could regulate cytokine production and the formation of autoantibodies (9, 10). Convergent pathways that specific interventions can target are found in RA, where the sustained absence of a particular metabolic response in synovial immune cells may perpetuate chronic inflammation and joint destruction (2).

While many studies have examined individual cytokines or autoantibody patterns in rheumatoid arthritis (RA) and systemic lupus erythematosus (SLE), several important questions remain unaddressed. Previous studies have used single-disease cohorts or immune markers from individual diseases to make direct comparisons among diseases and to capture patterns in the immune network associated with disease heterogeneity. In addition, most published biomarker studies have focused on diagnostic correlations rather than on immune system classification or on the application of the results to precision medicine (7, 11). While recent machine-learning-based analyses have shown that combined cytokine and autoantibody signatures can further stratify severity and immune heterogeneity in SLE (12), there has been limited understanding of comparative integrated immune-network analyses across RA and SLE within a common analytical framework. Furthermore, there is limited research that has taken a multidimensional approach to defining clinically relevant immune subphenotypes associated with disease activity and organ involvement, including cytokine correlations, autoantibody burden, principal component analysis, and unsupervised clustering. Thus, the novelty of the present study lies in its integrated cross-disease immune-network approach combining cytokine and autoantibody profiling in a large retrospective RA and SLE cohort to identify shared and disease-specific immune architectures relevant to disease stratification.

This study aimed to characterize immune network dysregulation in SLE and RA and to assess whether integrated cytokine and autoantibody profiling, using a common analytical framework, would provide insights into new clinically relevant immune subphenotypes beyond the classical serological classification. Our goals were to examine shared and disease-specific immune pathways, characterize multi-dimensional immune interactions through network-based analyses, and evaluate the translational relevance of integrated immune signatures for disease stratification.

## 2 Methodology

### 2.1 Study design and setting

Immunomodulatory reactions in rheumatoid arthritis (RA) and systemic lupus erythematosus (SLE) were studied alongside autoantibody-mediated inflammation, which was used to formulate the results of this retrospective cross-sectional observational study. The research was conducted in tertiary care hospitals and their affiliated diagnostic laboratories to access extensive immunological and clinical data. This study was conducted from January 2021 through December 2025, ensuring adequate temporal coverage to capture disease variability and treatment patterns. The methodological framework was informed by prior cytokine profiling and biomarker-based studies, while advancing toward a systems-level immune network approach.

### 2.2 Study population and sample size

The study involved 875 patients diagnosed with RA or SLE according to predefined classification criteria. RA was diagnosed according to the 2010 ACR/EULAR criteria (13), and SLE according to the 2019 EULAR/ACR criteria (14).

**Figure 1:**
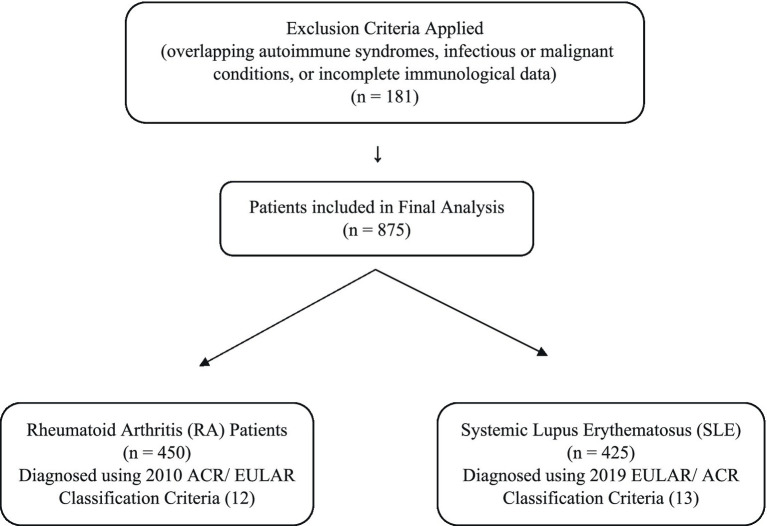
Participants selection flow diagram. Flow diagram of patient selection showing screening, exclusion criteria, and final cohort stratification into rheumatoid arthritis (RA) and systemic lupus erythematosus (SLE) groups.

Electronic medical records and laboratory databases initially identified 1,056 potential subjects. Patients were screened according to predefined inclusion criteria, including a confirmed diagnosis, availability of cytokine profiling data, documented autoantibody status, and complete clinical records. Following screening, 181 patients were excluded due to overlapping autoimmune syndromes, infectious or malignant conditions, or incomplete immunological data, in order to minimize confounding in immune biomarker analysis. After applying these criteria, 875 patients were included in the final analysis, comprising 450 with rheumatoid arthritis (RA) and 425 with systemic lupus erythematosus (SLE), as shown in Figure 1.

As the focus of this study was not to compare disease with non-disease states, no healthy control group was included; the analytical framework was tailored to detect cytokine signatures shared across autoimmune diseases and disease-specific immune patterns rather than to establish physiological ranges for cytokines. Although data were obtained from electronic medical records spanning 2021–2025, each patient contributed a single representative clinical and immunological dataset corresponding to a clinically defined disease activity assessment window. Therefore, no longitudinal repeated-measures analysis was performed, ensuring independence of observations and consistency of statistical modeling.

### 2.3 Clinical variables and data collection

A standard data abstraction protocol was used to extract data from institutional databases systematically. The demographic variables were age, sex, and disease period. Smoking history was defined as “ever smoking” (current or former smoking history documented in the medical record). Clinical parameters included disease activity indices, organ involvement, treatment history, and comorbid conditions, as well as laboratory data comprising standard hematological and biochemical parameters, inflammatory markers, and detailed immunological profiles.

Exposure to treatments reported comprised conventional synthetic disease-modifying anti-rheumatic drugs (csDMARDs), biologic drugs, steroids, antimalarials, and immunosuppressive drugs. The most commonly used RA medications in patient histories were methotrexate (7.5–25 mg/week), leflunomide (10–20 mg/day), sulfasalazine (1–3 g/day), TNF inhibitors, IL-6 inhibitors, and Janus kinase inhibitors. Therapies administered for SLE encompassed hydroxychloroquine (200–400 mg/day), corticosteroids (prednisone equivalent), mycophenolate mofetil, azathioprine, cyclophosphamide, belimumab, and rituximab. Due to retrospective multicenter variability, medication doses could not be standardized for the cumulative dose analysis.

Only data collected in stable clinical visits or specified disease activity states were used to ensure consistency. All extracted data were re-identified before analysis to maintain patient confidentiality.

### 2.4 Key variables and operational definitions

#### 2.4.1 Immune network dysregulation

Immune network dysregulation was defined as the disruption of immune homeostasis, characterized by an abnormal pattern of cytokine expression, the presence of pathogenic autoantibodies, and altered interactions among these elements.

#### 2.4.2 Cytokine signatures

The patterns of quantitative and relative expression of circulating cytokines that defined the states of immune activation were termed cytokine signatures. Those that promoted inflammatory responses were called pro-inflammatory cytokines, and those that engaged in immune regulation were called anti-inflammatory cytokines.

#### 2.4.3 Autoantibody profile

The presence and titers of disease-specific antibodies against self-antigens were defined as the autoantibody profile. Seropositivity was determined according to laboratory-specific references, and high-titer positivity exceeded clinically important thresholds.

#### 2.4.4 Disease activity assessment

Disease activity was defined as an indicator of disease severity at the time of assessment using validated composite indices for RA and SLE. Disease activity in RA was assessed using the Disease Activity Score in 28 Joints (DAS28) (15), categorized as remission (<2.6), low (2.6–3.2), moderate (3.2–5.1), and high (>5.1).

In SLE, disease activity was measured using the SLEDAI, categorized as mild (<6), moderate (6–10), high (11–19), and very high (≥20), with scores ≥10 considered clinically significant active disease (16).

For rheumatoid arthritis (RA), the DAS28 calculation included the patient’s global health assessment, ESR or CRP, tender joint count (28 joints), and swollen joint count (28 joints), based on the validated DAS28 methodology. In systemic lupus erythematosus (SLE), the SLEDAI assessment included evaluation of renal, hematological, mucocutaneous, musculoskeletal, serosal, neurological, vascular, constitutional, and immunological manifestations, according to standard SLEDAI definitions.

#### 2.4.5 Flare and treatment response

Therapeutic escalation was defined as a flare in SLE based on the following criteria: an increase in SLEDAI score of at least 4 points and/or the need for therapeutic escalation. This definition was derived from the existing flare definition used in SLEDAI and broadly aligns with clinically meaningful definitions of flares in the SELENA-SLEDAI Flare Index (SFI) framework, but formal categorization of the flares into mild/moderate and severe was not systematically available in the retrospective dataset. Treatment response in RA was defined as an improvement in DAS28 of ≥1.2 or a transition to low disease activity/remission, in accordance with the European League Against Rheumatism response criteria (17).

#### 2.4.6 Immune sub-phenotypes

The immune subphenotypes were defined as discrete groups of patients characterized by clustering of combined cytokine and autoantibody measurements, reflecting heterogeneous immune phenotypes.

#### 2.4.7 Precision medicine applicability

The applicability of precision medicine was determined by the possibilities of integrating an immune profile into individualized disease classification, prognosis, and therapeutic decision-making.

#### 2.4.8 Hypertension and diabetes mellitus

Hypertension was defined as documented physician-diagnosed hypertension, use of antihypertensive medication, or a blood pressure ≥140/90 mmHg recorded during routine clinical evaluation. Diabetes mellitus predominantly represented type 2 diabetes mellitus based on documented clinical diagnosis and treatment records; isolated cases of type 1 diabetes mellitus were uncommon and not separately analyzed.

### 2.5 Assessment of cytokine signatures

Serum cytokine levels were measured using validated multiplex immunoassay platforms or enzyme-linked immunosorbent assays, in accordance with institutional protocols. The cytokine panel included key pro-inflammatory, anti-inflammatory, and regulatory mediators implicated in the pathogenesis of autoimmune disease, such as tumor necrosis factor-alpha, interleukins (IL-6, IL-10, IL-17), interferon-gamma, and selected chemokines.

Blood samples were drawn from peripheral veins during routine clinical visits in accordance with institutional procedures. The samples were centrifuged at 1500–2000 × *g* for 10–15 min after collection and then stored in cryovials to minimize freeze–thaw cycles. The samples were kept frozen at −80 °C until analysis. Frozen samples were thawed on ice and gently mixed as per the manufacturer’s recommendations before assay procedures. Cytokine measurements were retrospectively obtained from participating tertiary-care hospitals and affiliated diagnostic laboratories. As cytokine testing had originally been performed as part of routine clinical care across multiple institutions between 2021 and 2025, complete information regarding assay manufacturers, reagent vendors, catalog numbers, immunoassay platforms, and reagent lot numbers was not consistently available for all archived laboratory records. Only cytokine measurements generated using validated clinical laboratory procedures and institutional quality-control standards were included in the study dataset. All cytokines included had inter- and intra-assay coefficients of variation (CVs) of less than 15%. All assays were performed with calibration controls and duplicate internal quality-control samples to reduce the analytical variability. Whenever possible, samples from the RA and SLE groups were run in the same analytical runs.

The data were collected from several affiliated centers using a retrospective approach. Due to the retrospective multicenter study design, cytokines could have been measured using different validated analytical platforms used in routine clinical care. Laboratory records were not always complete with detailed platform-specific information and reagent lot documentation. To minimize inter-laboratory variability, only measurements generated under accredited laboratory quality-control procedures were included. Data integration was preceded by distributional assessment and principal component analysis to assess batch effects and distributional differences. Cytokine values were standardized using z-score transformation according to the formula:


Z=(X−μ)/σ

Where X denotes the observed cytokine concentration, μ the mean cytokine concentration, and σ the corresponding standard deviation in the pooled study dataset. Standardization was performed to minimize scale-related variability while preserving relative biological differences between disease groups.

The selection of biomarkers was hypothesis-driven and based on prior evidence of the central role of these cytokines in immune dysregulation in RA and SLE. The panel included pro-inflammatory cytokines (TNF-*α*, IL-6, IL-17, and IL-1β), interferon-related signaling (IFN-*γ*), immune regulation and B-cell activation (IL-10 and IL-4), T-cell activation (IL-2), recruitment of leukocytes (MCP-1), and stimulation of myeloid cells (GM-CSF). These biomarkers were selected for their established, reproducible relationships with disease activity, organ involvement, therapeutic response, and interactions with immune networks in previous RA and SLE studies. While other cytokines, chemokines, and TNF-family mediators are also involved in RA and SLE pathogenesis, the present study investigated a targeted panel based on reproducible clinical relevance, commonality between the two diseases, and availability in retrospective datasets.

Cytokine concentrations were originally measured in pg./mL and are presented in Table 1 as median (IQR) values for clinical interpretation. Distributions of cytokines were not normal; therefore, descriptive statistics were obtained using nonparametric methods. The standardized z-scores were then used for integrated analyses, such as correlation network analysis, principal component analysis (PAC), clustering, and multivariable regression modeling. Unless otherwise specified, all multivariate and dimensionality-reduction analyses were performed using standardized cytokine values.

**Table 1:** Serum cytokine concentrations (pg/mL) and correlation with disease activity.

Cytokine	RA (pg/mL)	SLE (pg/mL)	Between-group p-value	RA correlation with DAS28, *r* (95% CI)	*p*-value	SLE correlation with SLEDAI, *r* (95% CI)	*p*-value
TNF-α	18.5 [12.3–26.7]	22.9 [15.6–30.4]	<0.01	0.46 (0.38–0.53)	<0.001	0.38 (0.29–0.46)	<0.001
IL-6	14.2 [9.8–20.1]	11.7 [7.5–17.3]	<0.05	0.48 (0.40–0.55)	<0.001	0.29 (0.20–0.38)	<0.001
IL-17	9.6 [6.2–14.8]	7.4 [4.9–11.2]	<0.01	0.42 (0.34–0.49)	<0.001	0.25 (0.16–0.34)	<0.001
IL-10	6.3 [3.8–9.5]	11.8 [7.2–16.4]	<0.001	0.18 (0.09–0.27)	<0.001	0.44 (0.36–0.51)	<0.001
IFN-γ	10.5 [6.9–15.1]	14.6 [9.3–20.7]	<0.001	0.22 (0.13–0.31)	<0.001	0.51 (0.44–0.58)	<0.001
MCP-1	132.5 [98.4–180.2]	210.6 [150.3–278.5]	<0.001	0.25 (0.16–0.34)	<0.001	0.52 (0.45–0.59)	<0.001
IL-2	8.9 [5.7–12.6]	6.1 [3.9–9.2]	<0.01	0.37 (0.29–0.45)	<0.001	0.19 (0.10–0.28)	<0.001
IL-4	4.2 [2.6–6.8]	7.5 [4.8–11.1]	<0.001	0.15 (0.06–0.24)	0.002	0.41 (0.33–0.49)	<0.001
IL-1β	7.8 [5.1–11.3]	6.4 [4.2–9.6]	<0.05	0.39 (0.31–0.47)	<0.001	0.21 (0.12–0.30)	<0.001
GM-CSF	3.6 [2.1–5.4]	4.8 [3.1–7.2]	<0.01	0.27 (0.18–0.36)	<0.001	0.33 (0.24–0.42)	<0.001

Values are presented as median [IQR]. Cytokine concentrations are reported as raw serum levels (pg/mL). Correlations were assessed using Spearman’s rank correlation coefficient (r) between cytokine concentrations and disease activity scores (DAS28 for RA and SLEDAI for SLE). Ninety-five percent confidence intervals (95% CI) were calculated using Fisher’s z-transformation. Statistical significance was defined as *p* < 0.05.

### 2.6 Autoantibody profiling

Standardized immunoassays were used to assess autoantibody status. Rheumatoid factor (RF) and anti-cyclic citrullinated peptide (anti-CCP/ACPA) antibodies were tested in patients with rheumatoid arthritis (RA). Isotype information, predominantly IgM, some IgG and IgA, and anti-CCP isotype, was recorded wherever available in institutional laboratory records, but was not consistently available in all centers and therefore was not analyzed separately in the stratification of statistical modeling. Other anti-modified protein antibodies (AMPAs), such as anti-carbamylated protein antibodies (anti-CarP) and anti-acetylated protein antibodies (AAPAs), were not widely available in retrospective laboratory datasets and were therefore not included in the present analysis.

Standardized immunological assays were also used to detect antinuclear antibodies (ANA), anti-dsDNA, anti-Sm, anti-RNP, anti-Ro/SSA, and anti-La/SSB antibodies. Anti-dsDNA and extractable nuclear antigen (ENA) antibodies were analyzed using standardized ELISA or immunoblot assays according to institutional laboratory procedures, whereas ANA testing was performed using HEp-2 cells. RF and anti-CCP antibodies were measured using nephelometric or ELISA-based assays routinely used in accredited diagnostic laboratories. All participating centers used manufacturer-recommended positivity thresholds and laboratory-specific reference ranges.

Participating laboratories routinely subjected their assays to internal quality-control procedures and calibration standards to ensure assay reproducibility. Autoantibody positivity and levels were noted based on laboratory-specific reference values. These biomarkers were chosen due to their reported involvement in cytokine-mediated immune dysregulation pathways in autoimmune disease and their diagnostic/pathological significance.

### 2.7 Integrated immune network analysis

To assess immune network interactions, a multi-step analysis of integrated cytokine and autoantibody datasets was performed. The relationships between cytokines, autoantibodies, and clinical variables were evaluated using Spearman correlation coefficients. Partial correlation analysis, adjusted for age and sex, was conducted using rank-based nonparametric methods to account for the non-normal distribution of soluble immune mediator data.

Hierarchical agglomerative clustering of variables with Ward linkage and Euclidean distance was used for unsupervised clustering. Clustering analyses included IL-6, IL-17, TNF-*α*, IFN-*γ*, MCP-1, IL-10, IL-4, IL-2, GM-CSF, RF, anti-CCP, ANA, anti-dsDNA, anti-Sm, anti-RNP, anti-Ro/SSA, anti-La/SSB, and autoantibody burden. Immune variables were transformed to a standardized z-score and visualized using a heatmap with hierarchical dendrograms.

The optimal number of immune clusters was determined through combined evaluation of the elbow method, silhouette coefficient analysis, and cluster stability assessment. A five-cluster solution demonstrated the best balance between within-cluster compactness, between-cluster separation, and biological interpretability. Cluster stability was evaluated by the bootstrap method (1,000 iterations), and consistency in cluster assignment greater than 80% was considered sufficient to determine reproducibility.

Principal component analysis (PCA) was used to reduce the dimensions. The most important variables in immune variability were identified based on loading coefficients, with cytokines and autoantibody burden selected as key variables for inclusion in the principal components. Network visualization methods were used to identify major hubs of immune dysregulation and to characterize interaction dynamics in RA and SLE.

### 2.8 Stratification and outcome measures

The primary outcome of the study was the identification of disease-specific immune phenotypes and their associations with disease activity, clinical manifestations, and immune dysregulation in rheumatoid arthritis (RA) and systemic lupus erythematosus (SLE). Secondary outcomes included evaluation of immune profiles associated with treatment response, characterization of high-risk flare phenotypes in SLE, assessment of relationships between immune signatures and organ involvement, and the applicability of integrated immune profiling for patient stratification within a precision medicine framework.

Patients were stratified according to cytokine dominance patterns, autoantibody burden (single versus multiple autoantibody positivity), and integrated immune subphenotypes derived from unsupervised clustering analysis. In RA, treatment response was defined as a reduction in Disease Activity Score-28 (DAS28) of ≥1.2 points or achievement of low disease activity/remission. In SLE, high disease activity or flare-related progression was defined as an increase in the SLEDAI score of ≥4 points or the need for therapeutic escalation. Comparative analyses of RA and SLE were conducted within a unified analytical framework to enable consistent cross-disease interpretation of immune signatures.

### 2.9 Statistical analysis

Statistical analysis was performed using SPSS version 26.0 (IBM Corp., Armonk, NY, United States), with additional analyses conducted in R for advanced modeling and visualization. Continuous variables were reported as mean ± standard deviation (SD) for normally distributed data and as median with interquartile range (IQR) for non-normally distributed data, whereas categorical variables were summarized as frequencies and percentages. The Shapiro–Wilk test was used to assess the normality of continuous variables.

As soluble immune mediator levels demonstrated non-normal distributions, non-parametric statistical methods were used for cytokine-related analyses. Between-group comparisons were performed using the Mann–Whitney U test, and Spearman rank correlation coefficients were used for correlation analyses. Independent-samples t-tests were applied only to demographic and clinical variables that were normally distributed. To account for scale-related heterogeneity and reduce model instability, cytokine and immune mediator variables were standardized using z-score transformation [Z = (X − *μ*)/*σ*] before multivariable regression, clustering, and principal component analysis (PCA). Hierarchical clustering was performed using Ward’s linkage method with Euclidean distance, and PCA scatter plots were generated from standardized cytokine and autoantibody variables, with each point representing a single patient observation.

Dimensionality reduction was conducted using PCA, and components with eigenvalues greater than 1 were retained for interpretation. Variables with the highest loading coefficients were identified as major contributors to immune variability. False discovery rate (FDR) correction using the Benjamini–Hochberg method was applied to account for multiple comparisons in cytokine and immune marker analyses. Statistical workflow implementation for clustering, PCA, and correlation-network visualization was performed using standard R packages, including stats, factoextra, pheatmap, and corrplot. Silhouette coefficient analysis was used for cluster validation, yielding a value of 0.5, indicating acceptable cluster separation.

Variables demonstrating significance in univariate regression analysis (*p* < 0.10) were entered into multivariate regression models to identify independent predictors of disease activity and immune stratification outcomes. Logistic regression models were used for categorical outcomes, such as immune subphenotypes, whereas linear regression models were applied to continuous outcomes, such as DAS28 and SLEDAI scores. All multivariate models were adjusted for age, sex, disease duration, BMI, smoking status, hypertension, diabetes mellitus, dyslipidemia, current biologic therapy use, and corticosteroid exposure because of their potential influence on cytokine expression and immune activation profiles.

Adjusted odds ratios (ORs) and 95% confidence intervals (CIs) were reported. Complete-case analysis was used when missing data were less than 5%, whereas multiple imputation methods were applied when missingness exceeded 5%. As each patient contributed a single observation, all analyses were conducted within a cross-sectional framework without adjustment for repeated measures or within-subject correlation. All statistical tests were two-tailed, and *p*-values <0.05 were considered statistically significant.

### 2.10 Ethical considerations

This research was approved by the Clinical Research Project Ethics Committee of Tianjin First Central Hospital (Approval Number: 2025N146KY). The institutional review board granted ethical approval for the study, which followed the principles outlined in the Declaration of Helsinki. Since the study was retrospective, informed consent was waived by the committee.

## 3 Results

### 3.1 Study population characteristics

The final study comprised 875 patients with systemic lupus erythematosus (SLE) and rheumatoid arthritis (RA). The majority of patients in the group were female, with SLE exhibiting a greater female preponderance than RA. While RA patients had longer disease durations and more concomitant metabolic problems, SLE patients were often younger at presentation.

The RA patients were older and had fewer female patients than the SLE patients (52.6 ± 11.4 vs. 39.8 ± 12.1 years and 72.3% vs. 86.5%, respectively, *p* < 0.001). The metabolic comorbidities burden showed a greater prevalence of hypertension (41.2/28.6, *p* < 0.01) and diabetes (22.5/14.2, *p* < 0.05) in RA patients, but systemic involvement, especially renal (36.7/8.5, *p* < 0.001) and dermatological (42.8/9.3, p < 0.001) manifestations were much more pronounced in SLE patients with similar distribution of disease activity (Table 2). Additional SLEDAI-associated manifestations evaluated included musculoskeletal, serosal, constitutional, vascular, neurological, and immunological domains, although only the most prevalent organ manifestations are presented in Table 2 for conciseness. All analyses were performed on one indexed dataset per unique patient; no repeated patient-level samples were included in the analytical cohort. The study design was cross-sectional in nature with one-time patient-level data extraction; no temporal trajectory analysis was conducted.

**Table 2:** Baseline demographic and clinical characteristics.

Variable	RA (*n* = 450)	SLE (*n* = 425)	*p*-value
Age (years, mean ± SD)	52.6 ± 11.4	39.8 ± 12.1	<0.001
Female sex (%)	72.3	86.5	<0.001
Disease duration (years, median [IQR])	6.8 [3.2–10.5]	4.1 [1.8–8.7]	<0.001
BMI (kg/m^2^)	27.4 ± 4.6	24.9 ± 4.1	<0.01
Smoking history (%)	34.8	18.6	<0.001
Hypertension (%)	41.2	28.6	<0.01
Diabetes mellitus (%)	22.5	14.2	<0.05
Dyslipidemia (%)	38.1	26.4	<0.01
Moderate disease activity (%)	35.6	32.1	0.42
High disease activity (%)	26.2	26.8	0.81
Remission/low activity (%)	38.2	41.1	0.33
Renal involvement (%)	8.5	36.7	<0.001
Hematological involvement (%)	12.1	29.4	<0.001
Dermatological involvement (%)	9.3	42.8	<0.001
Current biologic therapy (%)	38.7	24.5	<0.01
Corticosteroid use (%)	44.9	61.2	<0.001

**Figure 2:**
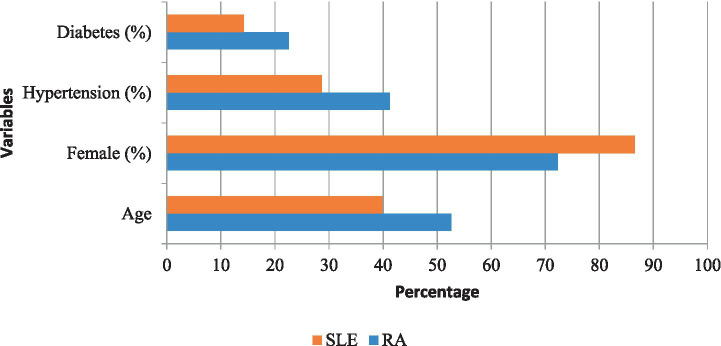
Baseline comparison (RA vs. SLE).

Values are expressed as mean ± SD, median (IQR), or percentages. *p*-values represent comparisons between groups. Figure 2 illustrates the demographic comparison of participants between RA (*n* = 450) and SLE (*n* = 425) groups. Current biologic therapy included TNF inhibitors, IL-6 inhibitors, B-cell targeted therapies, and other approved biologic agents according to institutional treatment protocols.

### 3.2 Cytokine signature profiles

SLE and RA showed different patterns of cytokine expression. SLE patients had higher levels of regulatory and interferon-associated cytokines than RA patients. As observed in Table 1, serum cytokine levels were measured in raw pg./mL units and then transformed into z-scores for multivariate analyses within the immune network, such as PCA, clustering, and regression modeling. Circulating cytokine analysis showed distinct, disease-specific cytokine patterns with RA patients having higher levels of IL-6 (14.2 vs. 11.7, *p* < 0.05) and IL-17 (9.6 vs. 7.4, *p* < 0.01), and SLE patients having higher levels of IL-10 (11.8 vs. 6.3, *p* < 0.001) and IFN-*γ*. Correlation analysis demonstrated that IL-6 showed the strongest association with disease activity in RA (*r* = 0.48, 95% CI: 0.40–0.55, *p* < 0.001), followed by TNF-*α* (r = 0.46, 95% CI: 0.38–0.53, *p* < 0.001) and IL-17 (*r* = 0.42, 95% CI: 0.34–0.49, *p* < 0.001). In SLE, MCP-1 (*r* = 0.52, 95% CI: 0.45–0.59, *p* < 0.001) and IFN-γ (*r* = 0.51, 95% CI: 0.44–0.58, *p* < 0.001) demonstrated the strongest correlations with disease activity, indicating a dominant role of interferon- and chemokine-mediated pathways in systemic immune dysregulation.

Standardized z-score transformed values were used for PCA, clustering, correlation network analyses, and regression modeling. A limited examination of the clinical records of the various institutions’ laboratory services also indicated that in clinically severe SLE patients, levels of various type I interferon-related biomarkers and selected chemokines were sometimes measured but not always and were therefore not included in the integrated modeling procedures.

Temporal coincidence: Cytokine measurements have been compared with clinical disease activity measures within a ± 2-week-interval to ensure similarity of immunological and clinical parameters between disease states. Pro-inflammatory cytokines and disease activity were more strongly correlated in RA, while regulatory and interferon-related cytokines were more strongly correlated in SLE. Distinct patterns of cytokine correlations were observed between RA and SLE.

### 3.3 Autoantibody profiles

Table 3 shows the autoantibody distribution, indicating that RA was characterized by strong disease specificity for RF (76.4%) and anti-CCP (71.9%), both of which were statistically significant (*p* < 0.001), whereas SLE patients exhibited a high prevalence of ANA (92.3%) and anti-dsDNA (68.9%). Notably, multiple autoantibody positivity was much more common in SLE (61.5% vs. 19.6%, *p* < 0.001) and was closely associated with disease severity and flare risk.

**Table 3:** Autoantibody profiles and burden analysis.

Autoantibody	RA (%)	SLE (%)	*p*-value	OR (95% CI)	Association with disease activity
RF	76.4	18.2	<0.001	14.2 (10.5–19.3)	Moderate (RA)
Anti-CCP	71.9	9.6	<0.001	24.8 (17.3–35.6)	Strong (RA severity)
ANA	28.7	92.3	<0.001	0.03 (0.02–0.05)	Weak
Anti-dsDNA	6.5	68.9	<0.001	0.04 (0.03–0.07)	Strong (SLE flares)
Anti-Sm	2.1	34.7	<0.001	0.04 (0.02–0.08)	Moderate
Anti-RNP	5.4	41.2	<0.001	0.08 (0.05–0.13)	Moderate
Anti-Ro/SSA	4.9	37.6	<0.001	0.09 (0.05–0.14)	Moderate
Anti-La/SSB	3.2	28.4	<0.001	0.08 (0.04–0.15)	Moderate
Multiple autoantibodies (%)	19.6	61.5	<0.001	0.15 (0.11–0.20)	Strong (SLE severity)

Values expressed as percentages of patients positive for each autoantibody. Odds ratios (ORs) are calculated using RA as the reference category; values <1 indicate a stronger association with SLE.

Only a few autoantibody data were available for subsets of SLE patients (anti-phospholipid-associated autoantibodies and lupus anticoagulant testing during routine clinical care). These were more commonly seen in patients with systemic/thrombotic manifestations but were not included in integrated clustering or regression analyses because they were not always available retrospectively and were subject to assay variability. Similarly, there was limited information on RF isotype in a few patients with RA, in which the predominant RF detected was IgM.

### 3.4 Integrated immune network and correlation analysis

Table 4 shows that correlation analysis revealed disease–specific differences in immune network architecture, with IL-6 being closely correlated with CRP in RA (*r* = 0.52) and IFN-*γ* being closely correlated with autoantibody burden in SLE (*r* = 0.56). MCP-1 demonstrated the strongest positive correlation with organ involvement in SLE (*r* = 0.52).

**Table 4:** Spearman correlation matrix of immune interactions in rheumatoid arthritis (RA) and systemic lupus erythematosus (SLE).

Interaction	RA Spearman r (95% CI)	SLE Spearman r (95% CI)	*p*-value
IL-6 – CRP	0.52 (0.45–0.58)	0.34 (0.26–0.41)	<0.001
IL-17 – DAS28/SLEDAI	0.42 (0.34–0.49)	0.25 (0.16–0.33)	0.004
IFN-γ – Autoantibody burden	0.21 (0.12–0.30)	0.56 (0.49–0.62)	<0.001
MCP-1 – Organ involvement	0.25 (0.16–0.33)	0.52 (0.45–0.58)	<0.001
IL-10 – DAS28/SLEDAI	0.18 (0.09–0.27)	0.44 (0.37–0.51)	0.006
TNF-α – Joint involvement	0.49 (0.42–0.55)	0.28 (0.19–0.36)	0.008
GM-CSF – DAS28/SLEDAI	0.31 (0.22–0.39)	0.35 (0.27–0.43)	0.021

r, correlation coefficient.

**Figure 3:**
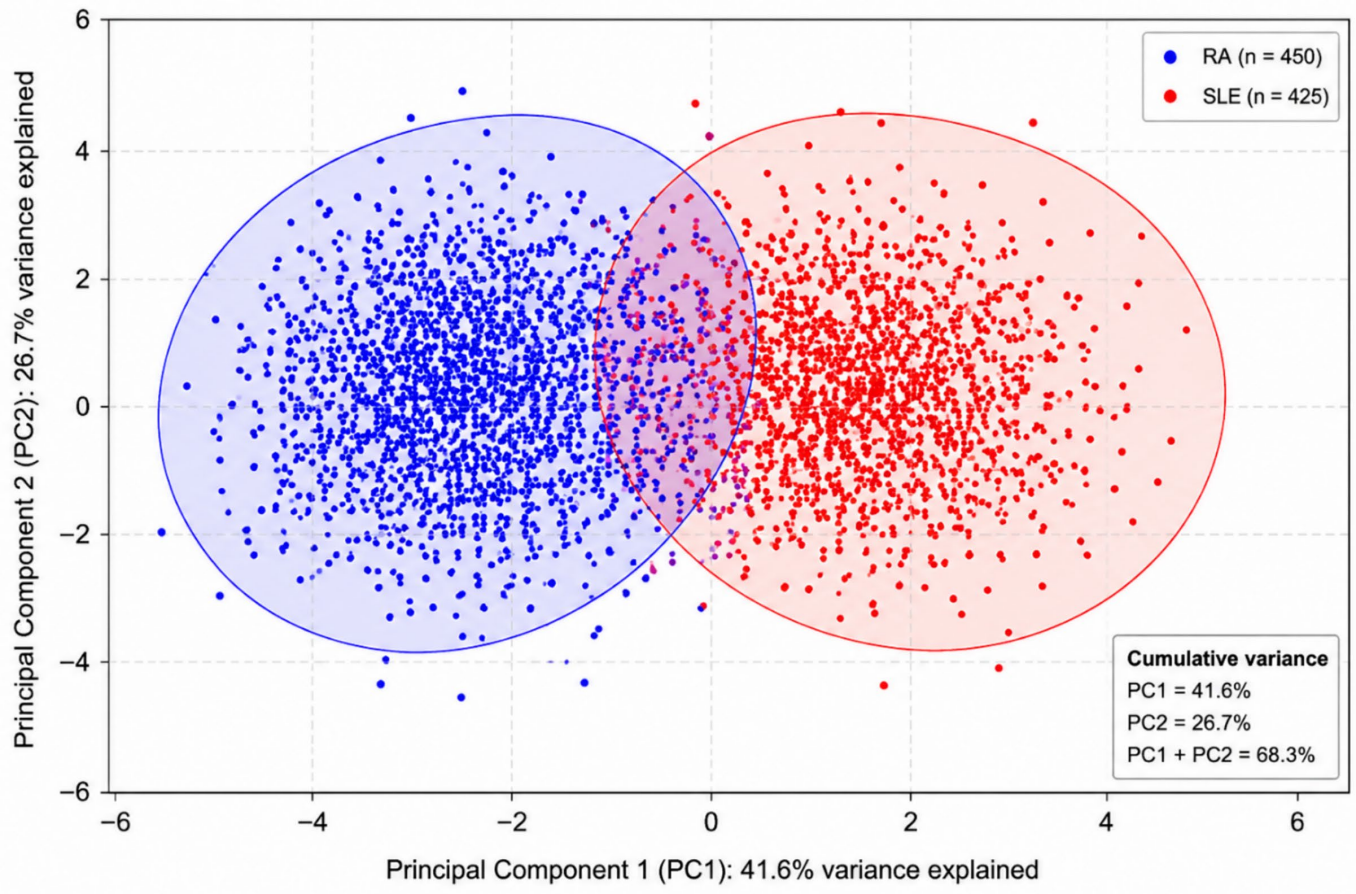
PCA scatter plot of RA and SLE patients based on cytokine profiles.

Principal component analysis (PCA) revealed that the first three principal components explained approximately 68% of the total variance in immune profiles. The primary contributors to this variance were IL-6, IFN-γ, MCP-1, and autoantibody burden, highlighting their central role in distinguishing immune network patterns between rheumatoid arthritis (RA) and systemic lupus erythematosus (SLE). These findings supported subsequent clustering analysis by identifying key variables driving immune stratification. Principal component analysis (PCA) was conducted to visualize the global variance in cytokine profiles. The PCA scatter plots of individual patient observations demonstrated partial separation between RA and SLE immune profiles along PC1 and PC2. PC1 and PC2 together explained the majority of the variance in immune profiles observed across the cohort (Figure 3).

Principal component analysis (PCA) scatter plot of rheumatoid arthritis (RA) and systemic lupus erythematosus (SLE) patients is based on standardized cytokine and autoantibody variables. Each point represents one individual patient. Principal component 1 (PC1) and principal component 2 (PC2) explain the largest proportion of immune-profile variance across the study cohort.

### 3.5 Immune sub-phenotypes and patient stratification

Five distinct immunological subphenotypes were identified across RA and SLE using unsupervised clustering of cytokine and autoantibody data. Selection of the five-cluster solution was supported by elbow plot inflexion analysis and maximization of the silhouette coefficient, indicating superior cluster separation and stability compared with alternative cluster solutions. Clinical outcomes, autoantibody burden, and cytokine dominance varied among these populations. Cluster 3 demonstrated increased standardized IFN-*γ* and IL-10 expression together with ANA and anti-dsDNA positivity, whereas Cluster 4 demonstrated elevated MCP-1 and IL-4 levels with ≥2 concurrent autoantibody positivities (Table 5).

**Table 5:** Immune sub-phenotypes identified through clustering analysis.

Cluster	% Patients	Dominant cytokine pattern	Autoantibody profile	Predominant disease	Clinical characteristics	Mean disease activity score	Secondary outcome
Cluster 1	24.6%	TNF-*α*/IL-6	RF+, Anti-CCP+	RA	DAS28 mean 5.4 ± 1.2	DAS28: 5.4 ± 1.2	TNF inhibitor response (OR 1.56, *p* < 0.01)
Cluster 2	18.3%	IL-17/IL-2	RF variable	RA	DAS28 mean 5.8 ± 1.4 with elevated IL-17 and IL-2 z-scores	DAS28: 5.8 ± 1.4	IL-17 blockade candidates
Cluster 3	21.5%	IFN-γ/IL-10	High ANA/dsDNA	SLE	SLEDAI mean 12.6 ± 3.1 with ≥2 flare episodes/year	SLEDAI: 12.6 ± 3.1	Steroid escalation prediction
Cluster 4	17.9%	MCP-1/IL-4	≥2 concurrent disease-associated autoantibodies	SLE	Renal and hematological involvement in >60% of patients	SLEDAI: 13.8 ± 3.6	Chemokine-targeted therapy
Cluster 5	17.7%	Mixed	Low burden	Both	DAS28 < 3.2 or SLEDAI <6	Low activity scores	Conservative therapy

Clusters were derived using integrated cytokine and autoantibody data.

**Figure 4:**
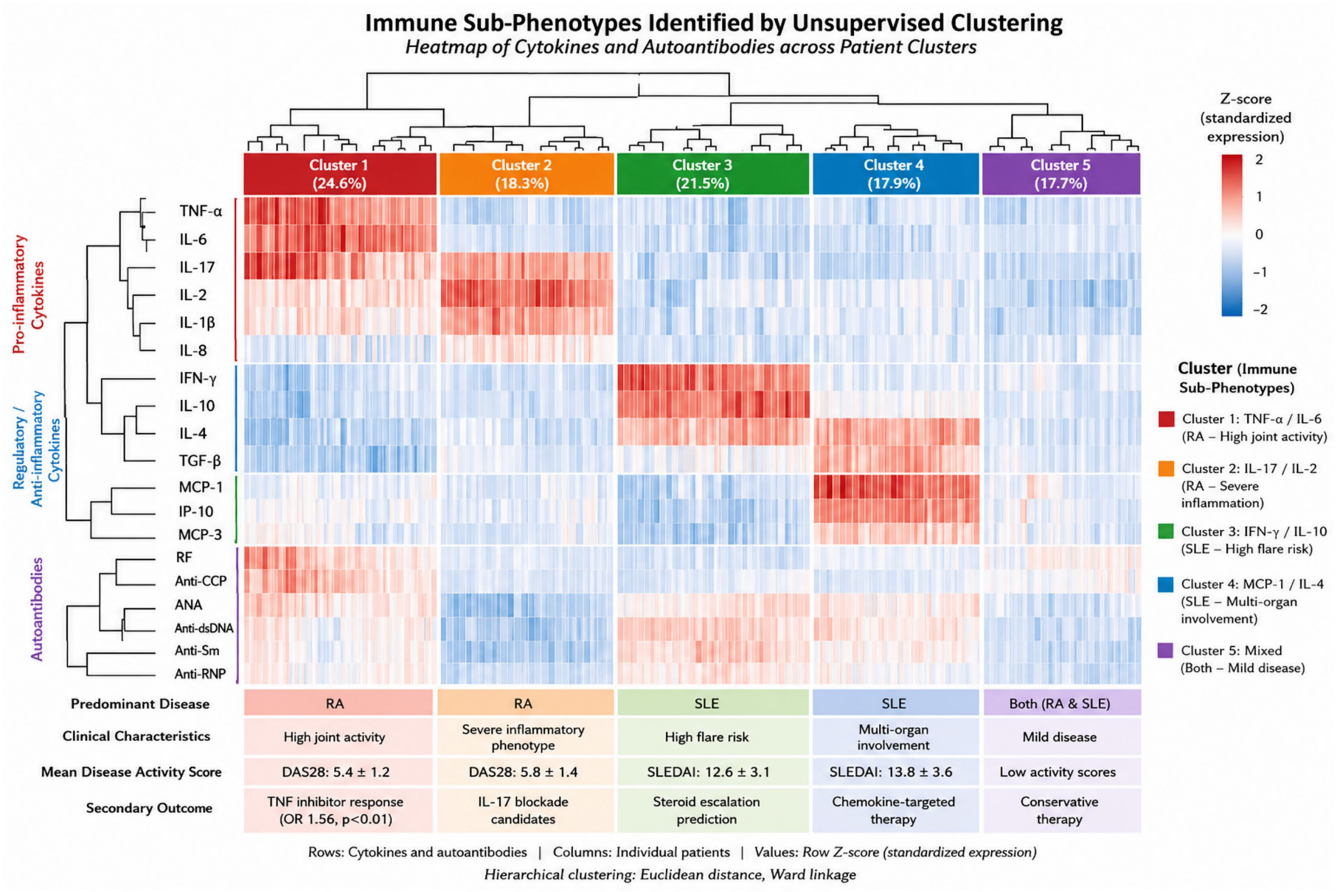
Hierarchical clustering heatmap of integrated cytokine and autoantibody immune profiles in RA and SLE.

The cluster validation analysis showed good cluster separation among the identified immune subphenotypes, with a mean silhouette coefficient of 0.62. The stability of the clustering model was established through a robustness and reproducibility check using cluster stability bootstrapped resampling, which was valid in more than 85% of iterations (Figure 4).

### 3.6 Predictors of immune dysregulation and clinical outcomes

In univariate analysis, IL-6, IL-17, IFN-*γ*, MCP-1, IL-10, GM-CSF, and autoantibody burden were significantly associated with disease activity outcomes. Following multivariate adjustment for demographic and clinical confounders, IL-6, IL-17, IFN-γ, MCP-1, IL-10, and GM-CSF remained independently associated with disease activity, whereas autoantibody burden was no longer statistically significant (Table 6). Sensitivity analyses, additionally adjusted for biologic therapy and corticosteroid exposure, demonstrated similar effect estimates for IL-6, IL-17, IFN-γ, and MCP-1, supporting the robustness of the cytokine-associated disease activity findings.

**Table 6:** Univariate and multivariate predictors of disease activity.

Variable	Unadjusted OR (95% CI)	*p*-value	Adjusted OR (95% CI)	*p*-value
IL-6	1.58 (1.31–1.90)	<0.001	1.42 (1.18–1.71)	<0.001
IL-17	1.44 (1.19–1.73)	0.002	1.35 (1.10–1.60)	0.006
IFN-γ	1.66 (1.39–1.98)	<0.001	1.51 (1.27–1.89)	<0.001
MCP-1	1.79 (1.48–2.13)	<0.001	1.63 (1.34–2.02)	<0.001
IL-10	1.36 (1.14–1.61)	0.004	1.29 (1.08–1.55)	0.009
GM-CSF	1.27 (1.06–1.51)	0.018	1.21 (1.03–1.46)	0.031
Autoantibody burden	1.24 (1.01–1.49)	0.047	1.18 (0.94–1.42)	0.080

OR, Odds ratio; CI, Confidence interval.

When cytokine-dominant immune profiles were used to predict disease activity and therapeutic response in a secondary outcome analysis, they were stronger predictors than autoantibody burden. Cytokine-based models demonstrated substantially greater predictive value compared to autoantibody-based models, although formal model discrimination metrics were not evaluated.

**Figure 5:**
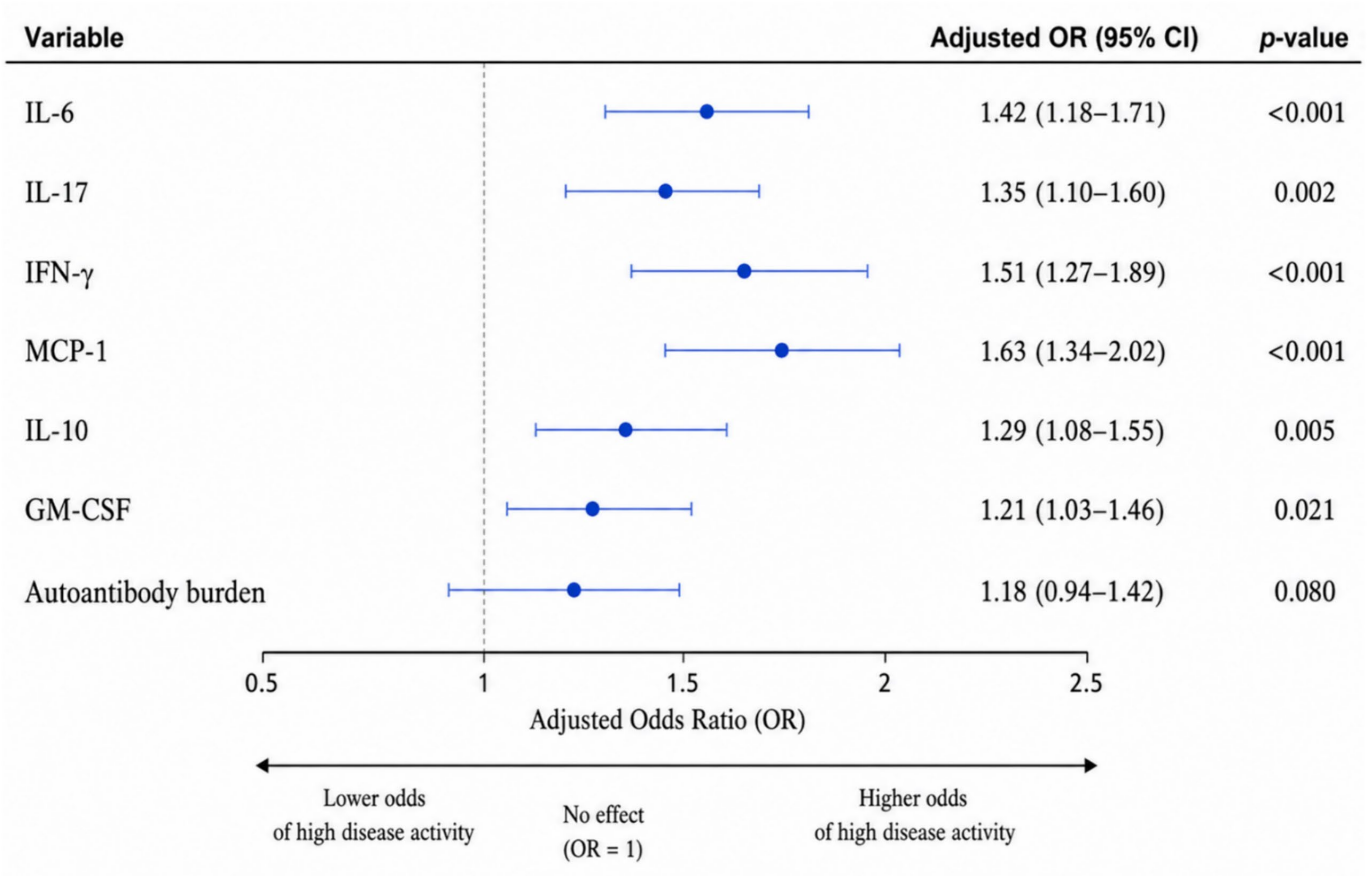
Forest plot of predictors for RA and SLE disease activity.

The forest plot (Figure 5) summarizes the odds ratios (OR) and 95% confidence intervals (CI) for key cytokines and autoantibodies as predictors of disease activity in RA and SLE, highlighting the most influential biomarkers. IFN-*γ* and MCP-1 demonstrated the largest adjusted odds ratios among SLE-associated immune mediators, whereas IL-6 and IL-17 demonstrated higher odds ratios among RA-associated cytokines.

Forest plot showing adjusted odds ratios (ORs) and 95% confidence intervals (CIs) for immune biomarkers associated with disease activity outcomes in rheumatoid arthritis (RA) and systemic lupus erythematosus (SLE). Each variable is plotted independently without connecting lines. The vertical reference line indicates OR = 1.

## 4 Discussion

This paper presents an in-depth analysis of immune network regulation in rheumatoid arthritis (RA) and systemic lupus erythematosus (SLE) based on demographic variables, metabolic and lifestyle variables, as well as cytokine and autoantibody profiles. The results reveal that, despite shared underlying immune malfunction, both diseases display distinct clinical and immunological phenotypes, which may be useful for disease stratification and biomarker development.

### 4.1 Study population characteristics

The demographic profile observed in the study is consistent with known epidemiological patterns. RA patients were older and demonstrated a lower female preponderance than SLE patients, consistent with reports that RA occurs more frequently with age and has a less pronounced sex disparity (18). In contrast, SLE was more prevalent in female patients and had an earlier age at onset, reflecting established sex-related immunological vulnerabilities (19).

RA was associated with longer disease duration, reflecting its chronic progressive nature and cumulative inflammatory burden. Long-term disease persistence has been linked with chronic immune activation and progressive tissue damage, which may influence clinical manifestations and biomarker profiles. RA patients were also more likely to exhibit metabolic comorbidities, including increased BMI, hypertension, diabetes, and dyslipidemia. These findings align with literature indicating that chronic systemic inflammation in RA is associated with metabolic imbalance and cardiovascular vulnerability (20). Smoking history was also more prevalent in RA, supporting its recognized role as an environmental contributor to immune dysregulation and autoantibody formation, particularly in seropositive disease (18).

Conversely, SLE demonstrated more extensive multisystem involvement, including renal, hematological, and dermatological manifestations. Such patterns are consistent with observational cohorts in which immune complex deposition contributes to widespread tissue injury (19). Despite these differences, disease activity distributions were not significantly different between groups, suggesting that distinct pathogenic mechanisms may converge to produce similar clinical disease burden in autoimmune disorders (20).

### 4.2 Cytokine signature profiles

Distinct cytokine signatures were identified between RA and SLE, indicating disease-specific inflammatory activation patterns. RA patients demonstrated elevated IL-6 and IL-17 levels, both of which were moderately correlated with disease activity. These findings support previous evidence linking Th17- and IL-6-mediated signaling with synovial inflammation and clinical activity in RA (21). Positive correlations were also observed between TNF-*α*, IL-1β, and disease activity, reflecting broader inflammatory cascades. Moderate associations between IL-2 and disease activity suggested the involvement of adaptive immune responses, whereas weaker correlations with GM-CSF may indicate a contributory role in inflammatory cell recruitment and activation (21).

In contrast, SLE patients demonstrated elevated IL-10, IFN-*γ*, MCP-1, IL-4, and GM-CSF levels, reflecting activation of interferon-, chemokine-, and humoral-associated pathways. IFN-γ and MCP-1 demonstrated the strongest associations with disease activity, while moderate associations were also observed with IL-10 and IL-4. These findings are consistent with previous evidence demonstrating coordinated cytokine–humoral immune activation in SLE, including relationships between IFN-γ, IL-17, anti-dsDNA, and anti-SSA antibodies (22). While both IL-10 and IL-4 have been considered regulatory cytokines, in the present study, they showed a positive correlation with SLE disease activity, indicating context-dependent immune-activating and B-cell-stimulatory roles in systemic autoimmunity (1).

The relatively weak relevance of cytokine-disease interactions in RA and SLE suggests that multiple immunological axes may be implicated in the pathogenesis of these diseases. Results indicate that the RA is more likely to have an imbalance in favor of pro-inflammatory cytokine pathways, and the SLE appears to have an imbalance of interferon/chemokine-associated pathways in immune activation, but there may be significant overlap and pathway dependence between these immune pathways. The same differences have been outlined in cytokine-based biomarker studies, in which specific immune patterns in disease were correlated with disease clinical phenotypes and remissions (23).

### 4.3 Autoantibody profiles

In this study, the autoantibody profiles in RA are different from those of SLE, suggesting basic differences in humoral immunity between the two diseases. The high prevalence of RF and anti-CCP antibodies in RA and their strong correlation with the presence and severity of the disease suggest that these antibodies are markers of antigen-driven immune responses and progression of the disease (5). Longitudinal studies also indicate that autoantibody patterns are present prior to the onset of clinical disease in RA and that they reflect the continual immunological dysregulation (24).

Anti-dsDNA, anti-Sm, and anti-ENA antibodies, however, were found in a high proportion of SLE patients. Anti-dsDNA antibodies are known to be associated with disease activity and organ involvement, and their high frequency in the present study is consistent with previous studies (25). The wide spectrum of autoantibodies found in SLE is thought to be due to the loss of immune tolerance and epitope spreading and is linked to the heterogeneity and disease course of SLE (26).

The odds ratios found in this study also support the discriminatory property of these biomarkers. There were strong associations with RA for RF and anti-CCP antibodies, and with SLE for ANA and anti-dsDNA antibodies, which have a high diagnostic and prognostic value in autoimmune diseases (5).

### 4.4 Integrated immune network and correlation analysis

A correlation study revealed disease-specific immune network design in RA and SLE. IL-6 was significantly positively correlated with CRP (*r* = 0.52), and IL-6 was at the hub of the inflammatory correlation network of joint pathology and systemic inflammation in RA. A moderate correlation was observed between IL-17 and RA disease activity (*r* = 0.42), consistent with its role in synovial inflammation (27). SLE showed strong correlations with autoantibody levels and IFN- *γ* and MCP- 1, which are important immune regulatory cytokines involved in interferon-gamma and chemokine-mediated immune responses, respectively. These are not independent downstream chemokine-signaling activities of IFN-*γ* since it can also induce the expression of other chemokines, such as MCP-1/CCL2, by signaling downstream of the others.

Correlations between IL-10 and disease activity (*r* = 0.44) further indicate that elevated IL-10 levels may reflect concurrent B-cell activation and immune amplification mechanisms in active SLE. Both diseases showed moderate associations with GM-CSF, suggesting that GM-CSF may be a common marker of immune activation. Principal component analysis (PCA) revealed that IL-6, IFN-γ, MCP-1, and autoantibody burden were the main variables explaining about 68% of the total variance in the immune profile. The results indicate that there are two immune networks, one in RA and the other in SLE, but they overlap to some extent with coordinated cytokine and humoral interaction (27, 28).

### 4.5 Immune sub-phenotypes and patient stratification

Unsupervised hierarchical clustering of integrated cytokine and autoantibody datasets identified five immunological subphenotypes across RA and SLE. RA was associated with clusters enriched in TNF-*α*/IL-6 and IL-17/IL-2 signaling patterns linked to increased joint inflammation (29, 30). These findings support the presence of inflammatory heterogeneity and suggest that coordinated cytokine patterns, rather than isolated mediators, are associated with distinct clinical manifestations.

In SLE, high autoantibody burdens clustered with interferon-related cytokines indicate coordinated humoral and cytokine-associated inflammatory pathways (4, 31). On the other hand, RA clusters were characterized by fewer autoantibody patterns and retained high levels of pro-inflammatory cytokines, which is consistent with targeted synovial immune responses (32).

Cluster validation demonstrated acceptable separation (silhouette coefficient 0.62) and stability (>85% in bootstrapped resampling), demonstrating the reproducibility of immune sub-phenotypes. These results indicate that integrated profiling will be able to identify immunologically unique patient groups with consequences for personal therapy choices and prognosis (29, 30).

### 4.6 Predictors of immune dysregulation and clinical outcomes

Multivariate regression showed dominant predictability of cytokine as a conditioned predictor of disease activity and severity compared to autoantibody burden. IL-6 and IL-17 (signaling pathways) were linked to RA activity and joint inflammation (23, 33), while IFN-*γ* and MCP-1 were linked to more severe disease involvement across the systemic disease. These cytokines are likely connected to immune pathways and not fully independent predictors of each other (34, 35). The moderate association between IL-10 and GM-CSF and disease activity in both diseases further supports their involvement in the regulatory feedback and inflammatory activation pathways.

Autoantibody burden by itself was not significantly predictive (*p* = 0.08), and beyond the evaluation of autoantibody burden, “integrated cytokine profiling” might offer further resolution to distinguish immune dysregulation and disease heterogeneity. Stratification based on cytokines was also consistent with secondary outcome measures and demonstrated a better capacity to predict treatment response and flare risk than classical autoantibody-based models (23, 34).

The present study goes beyond traditional marker disease association studies and incorporates cytokine signatures, autoantibody burden, PCA-based dimensionality reduction, correlation-network analysis, and unsupervised clustering under a single analysis. Previous studies have revealed individual immune mediators in RA and SLE, but not as many that have assessed the coordinated interactions of these mediators to identify clinically relevant disease-specific inflammatory patterns. The results confirm the promise of a multidimensional immune profile for novel identification of immune subphenotypes that are associated with the severity of disease, flares, and potential therapeutic targets.

The results from a translational point of view confirm the potential of biomarker-based stratification in a step-wise procedure. A clinically relevant panel of selected cytokines (IL-6, IL-17, IFN-*γ*, and MCP-1) and key autoantibodies (RA: RF/anti-CCP; SLE: anti-dsDNA/ANA) may help identify cytokine-dominant and autoantibody-dominant inflammatory phenotypes in routine clinical laboratories by multiplex immunoassay. These methods could facilitate individualized therapeutic and monitoring approaches, but need to be validated in prospective studies and standardized for assays to be put into practice.

In conclusion, the results support an overall role for the IL-6/IL-17 axis in RA and interferon- and chemokine-associated signatures in SLE. Another important finding of this study is that the cytokine and humoral interactions are not independent, but coordinated and interdependent. Differences in regulatory cytokines (IL-10) may be due to context-dependent immune functions, differences in disease stage, or patient stratification.

### 4.7 Strengths of the study

This retrospective cohort study of RA and SLE provides a systems-wide assessment of immune dysregulation, combining analyses of cytokine and autoantibody profiles. Immune network characterization increases robustness through the use of multi-dimensional methods of analysis, such as correlation analysis, principal component analysis, and unsupervised clustering. The validity of the findings is enhanced through standardization of cytokine measurements and confounder adjustments. Notably, the study has identified clinically relevant immune subphenotypes and demonstrated the greater predictive capacity of cytokines than autoantibodies, indicating their translational potential to advance precision medicine strategies.

### 4.8 Limitations of the study

This study has a retrospective design, which limits the ability to infer causal relationships and is likely to introduce selection bias, although it employs standardized inclusion criteria. Moreover, the study population was also selected from hospital-based cohorts, which may not be fully generalizable to the general population. Although normalization and standardization processes were used, heterogeneity in measurements of cytokines and autoantibodies could also have been caused by variability in laboratory platforms across centers.

Additionally, there was a lack of uniformity in retrospective laboratory records across all participating centers in terms of detailed data on assay manufacturers, immunoassay platforms, reagent vendors, catalog numbers, and reagent lot numbers. However, analytical variability across platforms could not be fully assessed; nevertheless, potential variability was minimized through standardization procedures and institutional quality-control requirements.

Furthermore, the cytokine and autoantibody panels were not comprehensive and included only biomarkers clearly available in retrospective institutional databases. Some of the mediators of the immune system that have been implicated in RA and SLE pathogenesis were not systematically evaluated, such as mediators of the interferon type (IFN-*α*/IFN-*β*), IL-1RA, IL-7, IL-15, IL-12p70, IL-21, IL-23, IL-5, IL-13, TGF-β, other chemokines (CXCL-family), other members of the TNF-family, and inflammatory mediators (osteopontin, resistin, stem cell factor). Similarly, more specific anti-modified protein antibodies (AMPAs), such as anti-carbamylated protein antibodies (anti-CarP) and anti-acetylated protein antibodies (AAPAs), as well as other SLE-associated autoantibodies, including anti-phospholipid antibodies, anti-ribosomal P, anti-chromatin, anti-SmRNP, lupus anticoagulant, and anti-β2-glycoprotein-I antibodies, were not necessarily recorded in the rear-view mirror. Furthermore, autoantibody isotype analysis was not available at all centers and was not always feasible to incorporate into integrated immune network modeling.

Moreover, single-time-point measurements limit the ability to measure time-dependent changes and variability in cytokine expression, which are also known to vary with disease activity and treatment exposure. Due to the retrospective study design, differences in the length and intensity of biologic therapy and corticosteroid use, and the potential for residual treatment-related confounding, these findings should not be viewed as definitive. In addition, the lack of extraneous validation cohorts restricts the generalization and reproduction of the identified immune sub-phenotypes. These limitations highlight the need for prospective longitudinal multicenter validation studies. Although standardized procedures and non-parametric analytical approaches were implemented, residual variability related to retrospective multicenter immune biomarker assessment, laboratory platform heterogeneity, and incomplete assay-level documentation cannot be completely excluded.

### 4.9 Future recommendations

Future studies should implement prospective longitudinal designs to evaluate temporal changes in inflammatory signatures and their relationship with disease progression and treatment response. Combining multi-omics technologies, such as transcriptomics and metabolomics, would provide further understanding of the immune-metabolic interactions. The generalizability of immune sub-phenotypes should be validated in other populations. To make these findings more clinically applicable, machine learning models can be incorporated, and predictive performance metrics can also be assessed. Finally, interventional research to study cytokine-specific subgroups is justified to support the translation of these findings into precise treatment plans.

## 5 Conclusion

This paper shows that RA and SLE have unique, yet partially similar, disease-specific cytokine dominance and autoantibody patterns that are a result of immune network dysregulation. The “integrated cytokine profiling” (based on IL-6/IL-17 and IFN-*γ*/MCP-1 pathway-associated signatures) was more closely linked to disease activity and clinical outcomes than autoantibody burden alone. These identified immune sub-phenotypes reflect the heterogeneity and interconnections among the autoimmune signaling networks. The results provide a more sophisticated comprehension of systemic autoimmunity, and they provide a framework for developing personalized treatment strategies.

The clinical implications of these findings include the potential to develop multiplex immune profiling panels combining cytokines and autoantibodies to enhance the stratification of RA and SLE patients. Such strategies could help to identify high-risk inflammatory phenotypes earlier and aid in more personalized therapeutic management. The clinical utility of these biomarkers, however, will need to be validated in prospective studies, using standardized assay thresholds and independent cohorts, before their incorporation into clinical practice.

## Data Availability

The raw data supporting the conclusions of this article will be made available by the authors, without undue reservation.
